# Intracerebral Borna Disease Virus Infection of Bank Voles Leading to Peripheral Spread and Reverse Transcription of Viral RNA

**DOI:** 10.1371/journal.pone.0023622

**Published:** 2011-08-22

**Authors:** Paula Maria Kinnunen, Hanna Inkeroinen, Mette Ilander, Eva Riikka Kallio, Henna Pauliina Heikkilä, Esa Koskela, Tapio Mappes, Airi Palva, Antti Vaheri, Anja Kipar, Olli Vapalahti

**Affiliations:** 1 Department of Veterinary Biosciences, Faculty of Veterinary Medicine, University of Helsinki, Helsinki, Finland; 2 Department of Virology, Haartman Institute, University of Helsinki, Helsinki, Finland; 3 Centre of Excellence in Evolutionary Research, University of Jyväskylä, Jyväskylä, Finland; 4 Department of Evolution, Ecology and Behaviour, Institute of Integrative Biology, University of Liverpool, Liverpool, United Kingdom; 5 Helsinki University Central Hospital, HUSLAB, Helsinki, Finland; 6 Finnish Centre for Laboratory Animal Pathology, Faculty of Veterinary Medicine, University of Helsinki, Helsinki, Finland; 7 Veterinary Pathology, School of Veterinary Science, University of Liverpool, Liverpool, United Kingdom; University of Kansas Medical Center, United States of America

## Abstract

Bornaviruses, which chronically infect many species, can cause severe neurological diseases in some animal species; their association with human neuropsychiatric disorders is, however, debatable. The epidemiology of Borna disease virus (BDV), as for other members of the family *Bornaviridae*, is largely unknown, although evidence exists for a reservoir in small mammals, for example bank voles (*Myodes glareolus*). In addition to the current exogenous infections and despite the fact that bornaviruses have an RNA genome, bornavirus sequences integrated into the genomes of several vertebrates millions of years ago. Our hypothesis is that the bank vole, a common wild rodent species in traditional BDV-endemic areas, can serve as a viral host; we therefore explored whether this species can be infected with BDV, and if so, how the virus spreads and whether viral RNA is transcribed into DNA *in vivo*.

We infected neonate bank voles intracerebrally with BDV and euthanized them 2 to 8 weeks post-infection. Specific Ig antibodies were detectable in 41%. Histological evaluation revealed no significant pathological alterations, but BDV RNA and antigen were detectable in all infected brains. Immunohistology demonstrated centrifugal spread throughout the nervous tissue, because viral antigen was widespread in peripheral nerves and ganglia, including the mediastinum, esophagus, and urinary bladder. This was associated with viral shedding in feces, of which 54% were BDV RNA-positive, and urine at 17%. BDV nucleocapsid gene DNA occurred in 66% of the infected voles, and, surprisingly, occasionally also phosphoprotein DNA. Thus, intracerebral BDV infection of bank vole led to systemic infection of the nervous tissue and viral excretion, as well as frequent reverse transcription of the BDV genome, enabling genomic integration. This first experimental bornavirus infection in wild mammals confirms the recent findings regarding bornavirus DNA, and suggests that bank voles are capable of bornavirus transmission.

## Introduction

Natural bornavirus infections are associated with chronic progressive neurological diseases: Borna disease virus (BDV) causes a classically fatal meningoencephalomyelitis mainly in horses [1], and avian Bornavirus (ABV) causes proventricular dilatation disease, which affects the autonomous nervous system in birds [2]. BDV infection can, however, also remain subclinical or result in mild neurobehavioral manifestations [1], [3]. Especially in humans, markers of BDV infection have been demonstrated in several neuropsychiatric diseases [4]–[6], but although BDV or a BDV-like agent appears to infect humans [7], the existence of human Borna disease is still debatable [8]–[10].

BDV is a neurotropic and noncytolytic RNA virus comprising, together with ABV, the family *Bornaviridae* in the order *Mononegavirales*. The viral RNA codes for six proteins, including the nucleo- (N) and phosphoproteins (P) [11], [12]. Surprisingly, since they have an RNA genome and are not retroviruses, extensive database searches have recently shown that bornaviruses, millions of years ago, integrated their genomic DNA counterpart into the genomes of primates and some other vertebrates [13], [14]. This sort of phenomenon has been demonstrated *in vitro* in some infected cell lines and also *in vivo*, in a laboratory mouse [13].

However, BDV or a BDV-like agent are not only endogenized, but also induce exogenous infections with associated diseases. Borna disease of horses is a long-known disease in central Europe, but is nowadays also occurring elsewhere, and occurs in several other vertebrates, for example the sheep, rabbit, dog, cat, and cow [1]. Instead of or in addition to an exogenous BDV infection, what cannot be excluded is whether possible human BDV-related symptoms [4] are linked to endogenous Borna-like sequences (EBL). The role of EBLs in human disease is unknown, and hypotheses exist as to both a protective role for them and a pathogenic function [13], [14].

Currently no reports exist on infection studies carried out in wild rodents. However, numerous laboratory animals can be experimentally BDV-infected [15]. Rats mainly develop fatal, non-suppurative encephalitis, when infected as immunocompetent adults, whereas neonatal infection leads to mild symptoms including locomotor hyperactivity, learning deficits, and abnormal social behavior, but with no evidence of an inflammatory response [15]–[19]. Neonatal infection in laboratory rats leads to abundant BDV excretion at least in urine [18], [20]. The literature on BDV infection of other laboratory rodents is less copious, but the golden hamster and certain mouse strains establish asymptomatic infections, whereas most mice of the MRL strain develop a fatal encephalomyelitis [15], [21], [22], and neonatally infected gerbils perish despite the lack of any pathological changes [23]. Still, what has been learned from infection studies in laboratory rodents, which often have altered susceptibility to infections due to deficits perhaps in innate immunity [24], [25], is not necessarily easy to generalize to any wild rodent species.

The epidemiology of BDV is still enigmatic. Should the results of the controversial circulatory immune complex-detecting method be confirmed [8], [10], the virus infects up to 100% of humans [26] and horses and would spread through the close contact of countless infected individuals. However, research groups using several other methods suggest a much lower prevalence [4]. Furthermore, numerous epidemiological data point towards the existence of a wild-life reservoir [27], [28]. For instance, in horses and sheep, the infection is not easily transmitted horizontally; BDV prevalence is higher on farms with poor rodent control and hygiene [29]; and BDV strains cluster geographically rather than according to species or year of isolation [30]. Furthermore, epidemics are observed at 2 to 5 year intervals [28], [29], and as for feline infections risk factors are their hunting and a rural habitat [31]. Moreover, BDV can be transmitted horizontally in laboratory rats via urine [20]. While these data provide indirect evidence of a possible reservoir in small wild mammals, natural infections have, indeed, been detected alongside probable equine Borna disease cases in small wild mammals: insectivores (the bicolored white-toothed shrew, *Crocidura leucodon*) in Switzerland have been BDV-positive based on the demonstration of BDV RNA and antigen in tissues, and voles (root/tundra vole, *Microtus oeconomus*; bank vole, *Myodes glareolus*) in Finland have harbored antibodies [32]–[34]. The bank vole is one of the most common rodent species in Europe [35] ― its distribution in Europe clearly includes the areas where BDV infections in animals are reported [27], [33], [36], [37].

Based on the epidemiological evidence of a wild rodent reservoir for BDV and our previous findings of BDV antibodies in bank voles, we hypothesized that bank voles can act as a BDV reservoir and could therefore be productively infected without overt pathology. Furthermore, because of the recent demonstration of integration of BDV and BDV-like DNA sequences [13], [14], we decided to test whether reverse transcription of exogenous BDV RNA into DNA occurs in these wild mammals during infection. We therefore infected neonate bank voles intracerebrally, monitored the voles for 2 to 8 weeks, and subsequently assessed the extent of BDV infection, the associated pathological changes, and the potential generation of BDV DNA. We found support for both hypotheses: a possible role for bank voles as a BDV reservoir or transmitter, and, after exogenous infection, *in vivo* reverse transcription of BDV RNA indicating that it represents a common phenomenon.

## Results and Discussion

### BDV replicates in the bank vole brain after neonatal intracerebral infection

In order to verify whether BDV infection can be established in bank voles, we inoculated newborn bank voles intracerebrally (i.c.) with 10^2^, 10^3^, or 10^4^ ffu of BDV [27] or phosphate-buffered saline (control voles) within 24 h of birth from BDV-negative dams. Litters including the dams were housed in separate, individually ventilated, HEPA-filtered cages, and young bank voles were euthanized 2, 4, 6, or 8 weeks post-infection (p.i.). Their brains were collected and examined by reverse transcriptase (RT) PCRs for BDV nucleocapsid (N) and phosphoprotein (P) genes [33], [38] and by immunohistology for the respective antigens [39], [40].

Both BDV RNA and antigen were detectable in all infected voles, whereas controls were BDV-negative (Table 1, Table S1), demonstrating productive infection. In one infected vole, no BDV-N RNA was detectable despite the presence of BDV N-antigen, which confirmed the productive infection. All other infected vole brains tested positive for the RNA and protein of both BDV-N and -P. Dams and controls remained negative until the end of the study.

**Table 1:** BDV antigen, RNA, and DNA in brain samples of infected and control bank voles at various time points after infection.

	2 weeks p.i.^a^	4 weeks p.i.	6 weeks p.i.	8 weeks p.i.	Total
**N and P antigen** b	10/10^c^	15/15	14/14	2/2	41/41 (100%)
**N gene RNA** d	10/10	14/15	14/14	2/2	40/41 (98%)
**P gene RNA** e	10/10	15/15	14/14	2/2	41/41 (100%)
**N gene DNA** d	5/10	11/15	9/14	2/2	27/41 (66%)
**P gene DNA** e	0/10	1/15	0/14	0/2	1/41 (2.4%)
**Negative controls, antigen, RNA and DNA** b **^,^** d **^,^** e	0/3	0/3	0/3		0/9 (0%)

a p.i. = post infection.

b BDV N and P antigens as detected by immunohistology [39], [40].

c Results expressed as number of positive samples/number of samples studied.

d BDV N gene as detected by RT-PCR (RNA) or PCR (DNA) [33].

e BDV P gene as detected by RT qPCR (RNA) or qPCR (DNA) [47].

### Intracerebral BDV inoculation leads to viral spread in the entire brain and the peripheral nervous system

**Figure 1:**
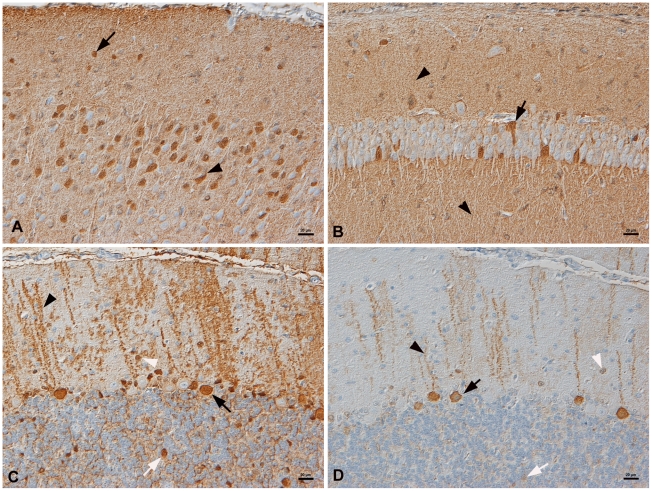
BDV antigens expressed in neurons in all brain areas. Bank vole 1. A.–C. Demonstration of BDV N protein with monoclonal antibody Bo-18. A. Cortex. A small proportion of neurons in the superficial granular layer (arrow) and numerous neurons in the superficial pyramidal layer (arrowhead) express viral antigen in cell bodies (arrowhead). The fine lined staining in the remaining parenchyma represents viral antigen in cell processes (see also B). B. Hippocampus, CA1. A few pyramidal cells (arrow) and scattered neurons in Stratum radiatum and Stratum oriens (arrowheads) express viral antigen C. Cerebellum. Purkinje cell bodies (black arrow) and processes (black arrowhead) exhibit the most prominent reaction. Some neurons in the granular layer (white arrow) and the molecular layer (white arrowhead) are also positive. D. Staining for BDV P protein shows a similar expression pattern, but with generally lower intensity. Arrows and arrowheads: see C. Bars = 20 µm.

To identify the BDV target cells and viral spread, immunohistology was employed using mono- and polyclonal antibodies for BDV N [39], [40] and P [40]. Viral antigen was detectable in a large proportion of neurons in all brain areas (cortex, hippocampus, hypothalamus, cerebellum, brain stem), and was detected both in cell bodies and processes (Figure 1). The expression patterns for both antigens were identical, but the nucleoprotein reaction was generally more intense (Figure 1), as would be expected in the acute phase of the infection when N is expressed more abundantly than P [41]. All neuronal cell types appeared infected, but in variable proportions. No obvious difference appeared in the distribution and intensity of viral antigen expression at the different time points p.i.

**Figure 2:**
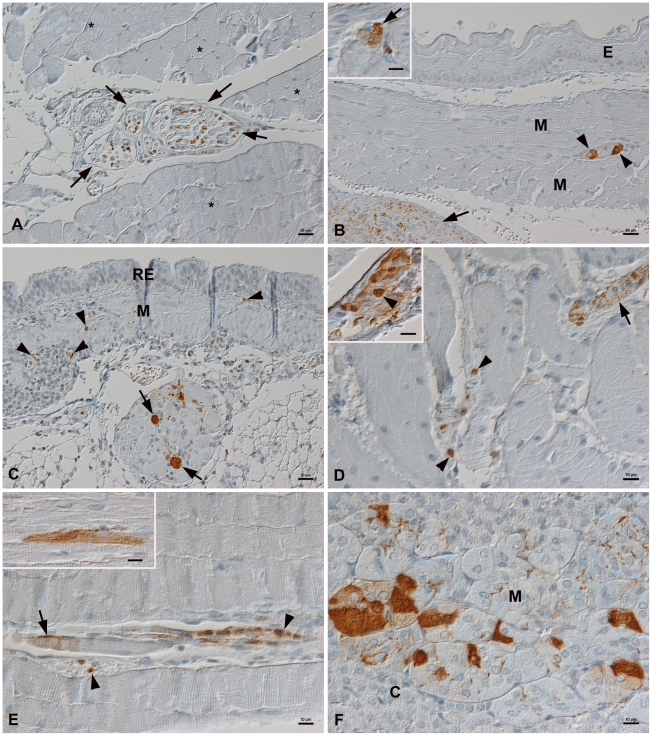
BDV antigen expressed in the peripheral nervous system. Demonstration of BDV N protein with monoclonal antibody Bo-18. A. Bank vole 5. Section of *M. quadriceps femoris* (*) with embedded femoral nerve (arrows) expressing BDV antigen in axons. Bar = 20 µm. B. Bank vole 4. Esophagus (E: epithelial layer). Between muscle layers (M) is a myenteric plexus structure with two neurons expressing BDV antigen (arrowheads). The adjacent mediastinal nerve exhibits abundant viral antigen in axons (arrow). Bar = 20 µm. Inset: B. Bank vole 4, expressing BDV antigen in neurons of a myenteric ganglion structure (arrow) in the urinary bladder wall. Bar = 10 µm. C. Bank vole 6. Trachea (RE: respiratory epithelium; M: muscle layer) and adjacent mediastinal ganglion with BDV antigen in neurons (arrows). Viral antigen is also expressed in axons of nerve fibers in the tracheal wall (arrowheads). Bar = 20 µm. D. Urinary bladder wall. Bank vole 36. BDV antigen is expressed in axons in an interstitial nerve (arrow) and in nuclei of smooth muscle cells (arrowheads). Inset: Bank vole 6. Smooth muscle cells express BDV antigen in both nucleus and cytoplasm (arrowhead). Bars = 10 µm. E, F. BDV antigen expression in non-neuronal cells. E. Bank vole 36. *M. quadriceps femoris* with BDV antigen in axons of interstitial nerve fiber (arrowheads) and in the cytoplasm of a myofiber (arrow). Inset: Bank vole 6. Myocardium with BDV antigen in a single myofiber. Bars = 10 µm. F. Bank vole 36. Adrenal gland. BDV antigen is expressed by several chromaffine cells in the medulla (M). C: Cortex. Bar = 10 µm.

BDV is known to spread centrifugally via peripheral nerves in experimentally infected rodents [42]–[45]. To assess whether this also occurs in bank voles, we examined a range of tissues for the presence of BDV antigen and observed it in axons in peripheral nerves, for example in the mediastinum and the mesentery, in skeletal muscle (femoral nerve), and in the urinary bladder in a large proportion (68%) of voles and as early as 2 weeks p.i. (Figure 2; Table S1). Neurons in autonomic ganglia (in mediastinum, esophageal wall, urinary bladder) were also infected (Figure 2B,C; Table S1). Interestingly, the urine of 3 voles whose interstitial nerve fibers in the urinary bladder wall exhibited viral antigen, tested positive for BDV RNA (Table 2). In 2 of these bank voles, a variable number of smooth muscle cells also expressed BDV antigen (Figure 2D; Table S1), which was seen in an additional 2 voles that did not appear to excrete virus into their urine at the time of euthanasia. These findings suggest that in bank voles, BDV spreads sooner than in mice (day 120 p.i.) [45], similar to the spread in newborn rats (day 14 p.i.) [43].

**Table 2:** Presence of BDV in urinary bladder, urine, and feces of experimentally infected bank voles.

Infectious dose	Study object	2 weeks p.i.^a^	4 weeks p.i.	6 weeks p.i.	8 weeks p.i.	Total
**10^2^ ffu**	Urinary bladder^b^	0/2^c^	1/3	1/3		2/8 (25%)
	Urine^d^	2/2	1/2	0/2		3/6 (50%)
	Feces^d^	0/1	1/3	0/3		1/7 (14%)
**10^3^ ffu**	Urinary bladder	0/2	2/5	2/4	0/1	4/12 (33%)
	Urine	0/2	0/5	0/6	0/1	0/14 (0%)
	Feces	0/2	3/4	5/6	1/1	9/13 (69%)
**10^4^ ffu**	Urinary bladder	0/2	4/4	4/4	1/1	9/11 (82%)
	Urine	0/4	0/6	2/5	1/1	3/16 (19%)
	Feces	0/5	4/6	5/5	1/1	10/17 (59%)
**All doses, total**	**Urinary bladder**	0/6 (0%)	7/12 (58%)	7/11 (64%)	1/2 (50%)	15/31 (48%)
	**Urine**	2/8 (25%)	1/13 (7.7%)	2/13 (15%)	1/2 (50%)	6/36 (**17**%)
	**Feces**	0/8 (0%)	8/13 (62%)	10/14 (71%)	2/2 (100%)	20/37 (54%)
**Negative controls** e	Urinary bladder	0/3	0/2	0/3		0/8 (0%)
	Urine	0/2	0/3	0/2		0/7 (0%)
	Feces	0/3	0/2	0/3		0/8 (0%)

a p.i. = post infection.

b BDV nucleocapsid (N) and phosphoprotein (P) antigens detected in urinary bladder by immunohistology [39], [40].

c Results are expressed as number of positive samples/number of samples studied (%).

d BDV N and P gene RNA detected in excreta by RT PCRs [33], [47].

e Negative control voles mock-infected with phosphate-buffered saline.

Apart from urinary bladder smooth muscle cells, other extraneuronal cells, such as the chromaffine cells in the adrenal medulla and myocytes in heart and skeletal muscle, occasionally expressed BDV antigen in voles with widespread expression in the peripheral nervous system (Figure 2E,F; Table S1), indicating peripheral nerves as the source of infection of parenchymal cells. All other tissues were BDV-antigen-negative. These findings are in accordance with BDV antigen detection in extraneuronal cells in a naturally infected horse [46], experimentally infected newborn rats and severely immunosuppressed adult rats [43], and recently, two naturally infected shrews [34]. Nonetheless, in all these animals including the voles in the present study, BDV antigen expression was mainly evident in neuronal tissues (reviewed by [1], [15]).

### Neonatally infected bank voles excrete BDV in urine and feces

BDV is known to be excreted in the urine but not in feces of neonatally infected rats [18], [20], [47]. Having demonstrated BDV antigen in nerves and ganglia of urinary bladder and the alimentary tract (esophagus), we examined the bank voles for BDV excretion in urine and feces by RT-PCR [33], [38]. Viral RNA was detectable both in urine (6 of 36; 17%) and in feces (20 of 37; 54%) (Table 2) of infected voles, but not in controls. The urine tested positive as early as 2 weeks p.i., but the feces were positive from 4 weeks on except for one euthanized symptomatic vole at 3 weeks (Table S1). The proportion of PCR-positive excreta increased with time up to week 6 p.i., most likely reflecting the time-span for centrifugal spread via peripheral nerves. Indeed, voles excreting BDV almost invariably expressed BDV antigen in their peripheral nerves (Table S1) and in particular in the urinary bladder, where interstitial nerve fibers tested positive in 60% (15 of 25) of the voles tested at weeks 4 to 8 p.i. (Table 2, Table S1).

Interestingly, BDV was detectable earlier in urine than in feces, although it did, overall, appear more often in the latter (Table 2). Such a difference in excretion kinetics may in part be related to an inhibitory effect of feces on RT-PCR. Results in hantavirus-infected bank voles (10 to 100 times fewer spiked hantavirus RNA copies/ml detectable in feces than in urine) suggest this [48]. If so, the fecal RNA prevalence is probably an underestimate, and excretion might occur also at the earlier time point. However, our results also indicate strong and long-lasting BDV excretion in urine, since BDV RNA was found in urine at all time points p.i. Similar to hantavirus in bank voles [48], BDV may be excreted intermittently in urine, because not all voles exhibiting BDV antigen in the bladder also showed BDV RNA in their urine at the time of sampling.

### Some, but not all, neonatally infected bank voles mount an antibody response to BDV

We utilized an immunofluorescence assay (IFA) [33] to study the antibody response of infected bank voles. BDV-specific antibodies were not detectable at 2 weeks p.i., only at 4 weeks p.i. and thereafter (Table S1). These results are similar to those from other experimentally infected rodents, in which antibodies were detectable from days 10 to 35 p.i. onwards, depending on rodent species, strain, immune state, and viral dose [47], [49]–[51]. Overall, antibodies were detectable in 41% (16 of 39) of the voles. Most (59%) voles, among them some tested after 6 weeks p.i., had no detectable antibody level. This phenomenon was once reported in one infected laboratory rodent [20] but is common in naturally infected animals such as cats, sheep, and horses [3], [40], [52]. The reason for the delay in or absence of a humoral response is unknown, but what must be considered is that our study animals were outbred and most likely had fully functioning immune systems – which is not necessarily the case in laboratory-bred rats and mice [24], [25]. This is supported by the fact that laboratory mice die soon after tick-borne encephalitis virus infection [53], whereas bank voles thrive despite infection [54].

Our data do suggest that, if based on seroprevalence, calculation of the true BDV prevalence in bank voles and number of voles shedding the virus would be an underestimation.

### BDV infection of neonatal bank voles induces only sporadic clinical and pathological changes

Each vole was clinically monitored on a daily and blinded basis and closely observed in an individual cage to detect any potential treatment-related symptoms. Then they were euthanized. The majority (31 of 41; 76%) of those infected and all 9 control voles remained free of clinical symptoms for the entire observation period; the remaining 10 voles (24%), however, developed neurobehavioral changes (Table S1). Four of these voles exhibited severe symptoms: One circled and fell down and had to be euthanized 3 weeks p.i., another occasionally showed tremor and was euthanized 6 weeks p.i., the third died after one day of locomotor hyperactivity, and the last one was first hyperactive and later atactic, tremoric, spastic, apathic, and emaciated, with scruffy fur (Table S1). The other 6 voles showed locomotor hyperactivity, unpredictably leaping, even jumping out of their cages, a finding similar to that in BDV-infected MRL/+ mice [50]. Female voles (7 of 16) were significantly more frequently affected than males (3 of 25; Mid-P exact test p = 0.0154), consistent with a study in rats that suggested a role for sexual hormones in Borna disease pathogenesis. However, the most relevant finding from our clinical examination was that BDV does generally not kill bank voles, but the infection is able to establish itself, and the virus to be shed for several weeks ― all important prerequisites for a reservoir.

The brains of all voles were examined histologically for pathological changes associated with BDV infection. Voles with clinical symptoms exhibited no evidence of an inflammatory reaction. The cerebrum, hippocampus, and brain stem were unaltered, as was the cerebellum in most (4 of 6) cases. The vole euthanized due to its clinical signs, however, showed reduced number of Purkinje cells (PC). Small numbers of disseminated PCs were undergoing necrosis or apoptosis or both, as confirmed by immunohistology for active, cleaved caspase-3 in occasional PCs ([55]; data not shown). This was despite the lack of apparent differences in viral antigen expression in comparison to that of other infected voles. In the vole that had shown an occasional tremor, its number of PCs also appeared lower than in controls.

Seven voles, which were euthanized 4 and 6 weeks p.i., had very mild focal leptomeningeal and occasionally adjacent parenchymal perivascular inflammatory infiltration consisting of mononuclear cells (macrophages, some lymphocytes) in the parietal cortex (Table S1). This was associated neither with clinical symptoms, morphological evidence of neuronal cell death, nor increased neuronal BDV antigen expression. Similar mild mononuclear infiltrations may appear in BDV-infected mice and neonatally infected rats independent of viral distribution [15], [56], [57]. What cannot be excluded is that such inflammatory infiltration was a response to the intracerebral injection. No other organ examined in any vole exhibited significant pathological changes.

Taking into account that the control voles remained asymptomatic, one can conclude that these neurobehavioral changes in infected voles were likely a consequence of BDV infection. However, based on sample size, these findings are not supported statistically (Mid-P exact, p = 0.055). Nonetheless, they are consistent with findings in neonatally BDV-infected laboratory mice and rats [15], [50], [58], which are considered to be a consequence of neurotransmitter imbalance [59]. Indeed, BDV seems to be able to alter and impair the functions of nerve cells through interference with the protein kinase C –dependent signaling by the P protein, affecting the stimulus-induced synaptic plasticity [60], [61]. Nevertheless, most voles in this study remained asymptomatic, and those symptoms observed did not significantly correlate with BDV infection status.

The clinical symptoms observed in the vole with evidence of PC death were dominated by circling and falling; symptoms probably resulting from the lack of PCs' inhibitory effect on the vestibular nuclei [62]. PC loss has also occurred in neonatally infected rats [63]. These findings provide evidence of a direct effect of BDV on PC. The precise mechanisms of PC loss are likely complex, but apoptosis apparently contributes to it [63]–[65]. Unlike in rats, BDV appeared to induce no neuronal cell death in experimentally infected gerbils, while inducing clinical symptoms, and, contrary to our findings, the neuronal BDV expression pattern differed between symptomatic and healthy gerbils [23].

As intracerebral infection induced only sporadic and mild pathological responses in our study, it can be concluded that BDV may not be a significant pathogen for bank voles.

### BDV RNA is reverse transcribed into DNA in bank voles *in vivo*

Recent studies demonstrate reverse transcription of BDV-like sequences and integration of the respective DNA into mammalian genomes [13], [14]. We were interested to know whether RNA from exogenous and consequently replicating BDV in bank voles is efficiently transcribed into DNA. We employed two PCRs without the RT step to amplify BDV N and P genes from infected voles' brain DNA [33], [38].

**Figure 3:**
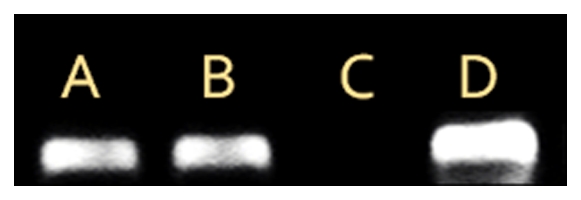
BDV RNA and DNA present in vole brain as verified with PCR and nuclease treatments. BDV DNA amplified by PCR without previous nuclease treatment (lane A) and after RNAse (lane B) but not after DNAse digestion (lane C). BDV RNA amplified with BDV RT-PCR (lane D).

BDV N gene DNA was present in 27 (66%), of those 41 infected, but not in the control voles, thus excluding amplification of possible endogenous BDV-like sequences (Table 1). BDV N DNA prevalence increased with increasing infection dose from 50% (4 of 8) to 78% (14 of 18), and was highest at 4 weeks p.i. at 73% (11 of 15; data not shown). This reverse transcription from RNA into DNA had occurred in half (5 of 10) of those voles studied as early as 2 weeks p.i. and was still detectable at 8 weeks p.i. In addition to the universal finding of N DNA, BDV P gene DNA was identifiable in one vole (Table 1, Table S1). This vole had received the highest dose and underwent testing at 4 weeks p.i. In addition, several other (12 of 41, 29%) infected voles showed a borderline result in the P-qPCR (Table S1). PCR positivity was sensitive to digestion of the template with DNAse, but not RNAse, confirming that detection of BDV DNA sequences was due to the presence of specific DNA and was not a result from nonspecific RNA amplification (Figure 3, Table 3). The sequenced 258-bp long N-amplicons from RNA RT-PCR and DNA PCR of one vole tested 6 weeks p.i. were 100% identical, with no sequence heterogeneity (quasispecies) observable (data not shown). These results verify BDV N-gene DNA findings from the extensive *in silico* studies [13], [14] and the more restricted experiments with cell cultures and 3 laboratory mice 30 days p.i. [13]: exogenous BDV N RNA is indeed reverse transcribed into DNA *in vivo* during infection. Furthermore, our study expands knowledge as to the time scale of the reverse transcription and adds data on P gene reverse transcription.

**Table 3:** PCR findings and verification of BDV DNA in four DNA-positive and one DNA-negative (*italicized*) bank vole.

Vole, code	Infectious dose, ffu	Weeks, post infection	P gene, qPCR^a^, C_t_ values		N gene, PCR^b^			
			RNA + RT	DNA	RNA + RT	DNA	DNA + DNAse	DNA + RNAse
**1**	10^3^	6	23.9	45.8	+	+	−	+
**9**	10^4^	4	20.8	46.7	+	+	−	+
**23**	10^4^	5	20.4	47.5	+	+	−	+
**40**	10^3^	6	20.6	47.2	+	+	−	+
***20***	*10^3^*	*6*	*18.8*	*No Ct*	*+*	*−*	*−*	*−*

a BDV P gene as detected by RT qPCR (RNA) or qPCR (DNA) [47].

b BDV N gene as detected by RT-PCR (RNA) or PCR (DNA) [33].

The mechanism of BDV reverse transcription in the bank vole remains to be elucidated. How and why BDV, but not some other RNA viruses, activates the reverse transcriptases, remains enigmatic, although BDV replicates in the nucleus. Likely candidates for reverse-transcribing BDV genes in bank voles are LINE-1 transposons (L1), which do exhibit reverse transcription activity [13]. Not only the human, but also the mouse and rat genome has included L1s ever since their common evolutionary ancestor [66], rendering it very likely that the bank vole also has these genes. Whatever the mechanism of reverse transcription in the brain of a BDV-infected bank vole, no mutations were detectable between the short BDV amplicons obtained from RNA and DNA.

Although this reverse transcription was consistent during bank vole BDV infection, further studies should investigate whether these viral genes exist in episomal DNA form or are inserted into the genome. The bank vole genome is not yet available to address directly the question whether endogenized BDV-like elements (EBL) exist in the bank vole genome, as demonstrated for some other rodents such as the rat, mouse, and squirrel [13], [14]. EBLs may play a role in the epidemiology of BDV, since they may be advantageous and enable a species to function as a reservoir. Species showing EBL sequences (like primates, shrews, mice, and rats) seem more resistant to severe or lethal bornavirus infection than those with none (horse, dog, cat, and rabbit) [14]. This may result from protection mediated by expression of indigenous BDV N or other components.

### Could the bank vole be a BDV reservoir?

The present study clearly demonstrates for the first time that one mammal, a wild rodent species very common in endemic BDV regions, the bank vole, can be infected with and excrete BDV, generally without developing clinical disease or overt pathological changes. More specifically, we were able to establish productive infection after intracerebral viral inoculation, based on the combined demonstration of viral RNA and antigen. That BDV is rarely lethal in the bank vole would fit well into the picture of a possible reservoir. Specifically, the reservoir species cannot be too severely affected to be able to spread a pathogen; benign behavioral alterations may even assist the spread.

The bank vole is very common in Europe [35], also throughout the areas of BDV distribution [27], [33], [36], [37]. Bank voles occupy many kinds of woody habitats, but in winter may reside inside animal sheds and homes [35]. Population densities fluctuate strongly, in a few-year cycle [67], [68]. Moreover, BDV case numbers have fluctuated in such cycles [28], [29], but any association with bank vole population densities remains to be shown. Interestingly, asymptomatic bank voles excrete and transmit another pathogen, Puumala hantavirus, in saliva, urine, and feces for at least 44 to 84 days p.i. with persistence of this virus [48], which has some similarities to BDV excretion in our findings.

Further experimental infections could be extended to include other inoculation routes and frequent urine, feces, and saliva sampling to characterize in more detail the pattern of BDV excretion, as well as to use cohousing with uninfected individuals to study transmission. In this study, no horizontal transmission of BDV from infected offspring to dams was detectable but cannot be excluded: Dams remained healthy, and no viral RNA, antigen, or antibodies could be detected at the end of the study, around 12 days after weaning and 40 days after infection of the pups. However, rat dams, after infection of their litters and when co-housed during the entire period, can acquire the infection and succumb to severe Borna disease between 3 and 5 months [47], suggesting that our observation period was too short to claim no horizontal BDV infection of the dams.

Based on the present results and the previous demonstration of BDV antibodies in wild-caught voles [33], a future study to identify wild, natural BDV carriers by RT-PCR and immunohistology would also be relevant.

### Conclusions

Bank voles can be productively infected after intracerebral inoculation of various doses of BDV. The infection does not generally lead to pathological alterations and is mainly subclinical. A minority of the infected voles produce antibodies. BDV infection in the bank vole is primarily neurotropic, although it spreads centrifugally from the widely infected central nervous system into several peripheral nerves and ganglia, for instance in the urinary bladder. Often, the virus is also excreted in urine and feces. Furthermore, BDV RNA is commonly reverse transcribed into DNA in bank vole brain tissue, verifying that this newly detected phenomenon, which is necessary for genome integration of sequences of an RNA virus, occurs readily *in vivo* during BDV infection. In addition to confirming this crucial step in the endogenization process, these data provide evidence that the bank vole can be a potential BDV reservoir.

## Materials and Methods

### Ethics statement

The County Administrative Board of Southern Finland approved the facilities and the protocol (Permit number ESLH-2006-03286/ym-23), which followed Finnish legislation for animal experiments (MMMa 36/2006). All efforts were made to minimize suffering.

### Animals, viruses and sampling

Thirteen serologically BDV-negative, pregnant laboratory-born bank voles of wild-caught parents, entered a Biosafety level 3 laboratory 1 to 2 weeks before giving birth to 2 to 6 pups each. Each litter lived, together with their dam, in an individually ventilated, HEPA-filtered cage (Isocage Unit, Tecniplast, Italy). We checked the function of the cage unit and the welfare of the voles daily. In addition to the usual forage and water, the voles ate raw potatoes to guarantee their fluid balance. The 41 newborn voles were infected intracerebrally (i.c.) with a 25G needle with 5 µl of fourth rat passage of the BDV/He80 strain, the so-called “rat BDV” [27] diluted in phosphate-buffered saline (PBS) to contain 10^2^, 10^3^, or 10^4^ ffu of virus, or, as a control (9 pups), with pure PBS. The voles were weaned at age of 4 weeks by removal of the dams. After 2 (13 voles), 4 (17 voles), 6 (16 voles) or 8 (2 voles) weeks post infection (p.i.), the voles were euthanized under isoflurane anesthesia by cervical dislocation. One severely symptomatic vole was euthanized at 3 weeks p.i., and another died 5 weeks p.i. Voles from each litter were equally included in groups at pre-set times. Before anesthesia, we observed the voles in their individual cages, followed by collection of blood samples from the retro-orbital sinus with capillary tubes under anesthesia just before euthanasia. Subsequently, urine and the rectum with feces (at least four times the volume of rectal tissue) and a range of tissues were aseptically collected, including brain, salivary glands, heart, lung and mediastinum, liver, kidney, spleen, urinary bladder, inner genitals, and *Musculus quadriceps femoris*, were stored at −80°C, or fixed in 10% buffered formalin at room temperature for one week or both, followed by routine paraffin embedding.

### Tissue homogenization and nucleic acid extraction

Brain tissue and feces (50–100 mg) were homogenized in 1 ml of Tripure Isolation Reagent (Roche) with 5-mm glass beads (LENZ Laborglas) and sterile sand (Merck) by 5000 rpm on the MagNAlyser homogenizer (Roche) for 45 sec. After centrifugation at 3000 g for 5 min, the supernatant was subjected to RNA extraction. For 50 to 100 µl of urine, RNA was extracted with 1 ml of the Tripure reagent; for smaller available volumes of urine, the amount was 500 µl. RNA findings were confirmed and DNA existence studied from brain tissue samples homogenized similarly in 1 ml of extraction buffer of the AllPrep RNA/DNA kit (Qiagen), and further processed according to manufacturer's instructions.

### PCRs and the verification of DNA findings

Urine and rectal/fecal RNA was reverse transcribed and the BDV nucleocapsid protein (N) gene amplified with nested PCR as described previously [33]. Brain RNA was processed in the same way but with a single, outer PCR round. The BDV phosphoprotein (P) gene RNA was detected by real-time qPCR as described [38]. BDV DNA was detected with the same N and P primers and probes in the same conditions but without the reverse transcription (RT) step. Only samples positive for both N and P RT-PCRs were interpreted as containing BDV RNA.

Amplicons originating from both RNA and DNA of one vole were purified with Exonuclease I and SAP enzymes (Fermentas) and cycle sequenced for both directions with Big Dye Terminator reagents (Applied Biosystems) in the ABI 3130xl capillary sequencer device. The sequences were checked and analyzed with the BioEdit program (http://www.mbio.ncsu.edu/BioEdit/page2.html).

DNA findings were verified by nuclease digestions: Extracted DNA was digested with DNAse for 30 min at room temperature followed by DNAse inactivation with 10 mM EDTA at 65°C for 10 min. As a positive control, similar digestion with RNAse was performed. Both enzymes originated from the RecoverAll kit (Ambion).

### Histology and immunohistology

Sections (3–5 µm) were prepared and stained with hematoxylin-eosin for histological evaluation, or were subjected to immunohistology. Immunohistology for the demonstration of BDV antigens was performed with the Ventana DAB biotin avidin detection kit on the Ventana Discovery Automatic Slidestainer (Ventana Medical Systems). The protocol included 20 min incubation with either rabbit polyclonal anti-BDV-nucleocapsid protein (N) 1∶5000 [40], mouse monoclonal anti-N antibody Bo-18 1∶100 [39], or polyclonal anti-BDV-phosphoprotein (P) 1∶20 000 [40]. BDV-infected horse brain tissue served as a positive control for all three anti-BDV antibodies. Consecutive sections incubated with the pre-immune serum instead of the anti-BDV antisera served as negative controls. Cleaved caspase-3 expression was demonstrated according to an earlier protocol [55].

### Serology

BDV-specific antibodies were sought from a 1∶10 PBS dilution of the whole blood samples with an immunofluorescence assay using BDV He/80 as the antigen in persistently infected C6 cells as described [33].

### Statistical methods

All the statistics were performed with an epidemiologic calculator in the Internet [69]. As recommended for small data sets [70], we employed the Mid-P exact test for analyzing significance.

## Supporting Information

Table S1 **Individual information of experimentally BDV-infected bank voles.**
(XLS)
